# Are low and high utilization related to the way GPs manage their practices? An observational study

**DOI:** 10.1186/s12875-018-0732-7

**Published:** 2018-04-25

**Authors:** W. A. de Graaf-Ruizendaal, L. van der Hoek, D. H. de Bakker

**Affiliations:** 1 NIVEL: Netherlands Institute for Health Service Research, PO Box 1568, 3500 BN Utrecht, The Netherlands; 2 Scientific centre for care and welfare, Tranzo, Tilburg University, PO Box 90153, 5000 LE Tilburg, The Netherlands

**Keywords:** Delivery of health care, Needs assessment, Regional health planning, General practice care, Utilization, SAE method

## Abstract

**Background:**

General practice care plays a key role in keeping healthcare effective and cost-efficient. However, variation in the utilization rates of practices may reveal variation in practice performance. Our research goal is to investigate whether the socio-demographic profile of the patients’ area of residence and practice organization characteristics influence the low or high utilization of general practice care.

**Methods:**

Data on the utilization of general practice care were derived from the electronic health records of 232 general practices participating in the NIVEL Primary Care Database for the year 2013. Census data for the year 2013 were matched with the postal code of the patients. A small area estimation (SAE) technique was used to calculate the estimated utilization rate for general practice care per practice based on the socio-demographic profile of the patients’ area of residence. Subsequently, the actual utilization rates were compared to the estimated rates per practice. Linear regression analysis was used to link the differences between the actual and estimated utilization rates to practice organization characteristics.

**Results:**

The socio-demographic profile of the patients’ area of residence accounted for 25.7% of the estimated utilization rates per practice. Practice organization characteristics accounted for 19.3% of the difference between the actual utilization rates and the estimated rates. Practices had higher utilization rates than estimated when a practice was a dual practice, when it employed female GPs, when it employed other healthcare providers and/or when it offered more services related to a disease management programme.

**Conclusion:**

We found that utilization rates of general practice care can be partially explained by the socio-demographic profile of the patients’ area of residence, but also by practice organization characteristics. Insight into these factors provides both GPs and the other stakeholders involved in the organization of general practice care with information to help reflect on the utilization of care.

## Background

General practice care plays a central role in healthcare. In the Netherlands, the general practitioner (GP) has a strong position: every resident is listed with a GP, and the GP deals with most of the health problems and coordinates the referral to specialized care [1]. Moreover, general practice care is covered by compulsory insurance and is the only health service exempt from cost sharing, which makes it accessible to everyone [2]. Many European countries regard strengthening the role of primary care, particularly general practice care, as a way to combat high healthcare costs and to meet the complex healthcare needs of the population [3–6].

Given the vital position of general practice care, it is important to study variation in utilization rates among general practices and especially the contributors to low and high utilization. Low utilization may not only indicate poor access and thus poor performance, but it could also indicate efficient use of healthcare services [7]. High utilization rates may indicate both good access and good performance, as a larger part of care is handled by the GP and less is referred to other healthcare providers. However, high utilization rates may also indicate inefficient use of healthcare services.

In the literature, the concepts underuse and overuse of healthcare services are often studied [8–11]. Underuse is ‘the lack of provision of necessary care’ and overuse is ‘the provision of care for which harms outweigh the benefits’ [9, 11]. To assess underuse and overuse, it needs to be determined whether the use of care was appropriate, i.e. whether the provision of medical services was beneficial or whether it had no or little benefit [11]. However, such methods are both time-consuming and expensive [10].

In the present study, we do not focus on the appropriateness of general practice care, but rather on high or low utilization rates at general practice level. High or low utilization rates at practice level were determined by the comparison of the actual utilization rates with the estimated rates based on the socio-demographic profile of the patients’ area of residence. From the literature, it is known that the socio-demographic characteristics of an area influence the use of general practice care [12]. For instance, areas with a high underprivileged index: a high percentage of the elderly living alone, children under 5 years, in single parent families, unskilled workers and unemployed adults were more likely to report a GP consultation [13].

However, differences are to be expected between the actual utilization rates of general practice care and the estimated rates based on a limited set of socio-demographic variables, because many more factors do influence healthcare utilization. We want to explore to what extent differences between the actual utilization rate and the estimated rate based on a limited set of socio-demographic variables can be related to practice organization characteristics. From the literature, it is know that service provision or general practice characteristics influence consultation rates [12]. For example, variation in frequent attendance in general practice care could for 3% be attributed to the age of the GP and the use of an appointment system [14]. Cervical smear uptake rates were substantially higher in practices with a female partner [15], and the number of partners in a practice positively influenced breast cancer screening rates [16]. Practice organization characteristics include the human resources available, specific services offered (e.g. diagnostic equipment), organization of consultation hours, participation in disease management programmes, physical accessibility of the practice and information dissemination.

In the present study, we investigated to what extent practice organization characteristics, next to socio-demographic characteristics at the four-digit postcode level, influence the utilization of general practice care. Our goal is to provide stakeholders who are involved with the organization of general practice care with useful insight into the practice organization characteristics that influence high or low utilization, to help them reflect on the organization and the utilization of general practice care.

## Methods

### Study design

An observational study was conducted in which for each practice the actual utilization rates of general practice care, based on medical record data, were compared with an estimated utilization rate for this practice, based on the socio-demographic profile of the patients’ area of residence. Using an SAE technique, estimates were made for every four-digit postcode area in the dataset, and based on these estimates, practice estimates were calculated as a weighted average of patients’ postcodes. The difference between the actual utilization rates and the estimated rates based on the socio-demographic profile of the patients’ area of residence were linked to practice organization characteristics using linear regression analysis. Furthermore, practices with the largest positive difference were compared with the largest negative difference using t-tests and proportion tests.

### Data

#### Utilization rates

Utilization rates of general practice care were derived from medical record data obtained from routine electronic health records from 240 general practices in the Netherlands participating in the NIVEL Primary Care Database for the year 2013 [17]. The database contains the utilization records, gender, age and four-digit postcodes of approximately 1 million listed patients who are representative of the Dutch population regarding gender and age. We selected general practices which had registered for a complete year and selected patients who had been registered at the same general practice for a complete year. This resulted in a dataset of 851,891 patients and 232 general practices, because 8 practices were excluded.

#### Practice organization characteristics

Practice characteristics were derived from the NIVEL database of health professionals (2013), which contains the characteristics of every general practice in the Netherlands (*n* = 5008) and which annually collects its data by means of surveys. Per practice, items were selected related to practice organization, such as type of practice (solo, dual, or group practice), human resources in practice (regarding GPs: number, FTE GP, age, gender, number of self-employed GPs, employed by another GP and locums, and the availability of different care providers in practice), list size (number of patients in practice) and number of patients in practice per FTE GP. Urbanization level of the practice was determined and divided into five categories from rural (less than < 500 addresses per km^2^) to very strongly urbanized (more than > 2500 addresses per km^2^). Subsequently, items were selected from the survey and categorized into five practice organization measures, regarding the number of medical services offered, consultation profile of the practice, physical accessibility of the practice, participation in disease management programmes, and the availability of patient information material. A higher score for a category refers to a higher occurrence of the category. The description of the measures and the items are presented in Table 1.

**Table 1:** Presence of the practice organization measures and underlying items for the sample of general practices (*n* = 232)

Measures	Items	%	Mean	SD
Medical service profile	Sum score of the 9 underlying items		2.99	1.50
*The practice offers the following medical services:*	Deliveries	1.3		
	Pharmacy	3.9		
	Minor surgery	89.7		
	ECG equipment	50.4		
	Spirometry	81.5		
	Audiometry	44.4		
	Teleconsultation	22.4		
	Medical equipment	0.9		
	Other medical services	4.3		
Consultation profile	Sum score of the 7 underlying items		4.11	1.54
*The practice offers the following consultation types:*	Scheduled consultations	91.8		
	Walk–in hours	13.8		
	Call back consultations	76.7		
	Evening consultations	15.1		
	Email consultations	47.4		
	Emergency line	89.2		
	Prescription line	77.2		
Accessibility	Sum score of the 3 underlying items		1.44	0.75
*The practice offers the following:*	Parking for disabled people within 100 m	64.7		
	Bus stop within 300 m	78.5		
	Other accessibility services	0.4		
Disease management	Sum score of the 14 underlying items		4.18	3.25
*The practice offers special consultation hours for the following diseases/categories:*	Diabetes	79.3		
	Asthma	23.7		
	COPD	34.1		
	Cardiovascular diseases	62.1		
	Minor surgery	45.7		
	Pre- and post-natal care	2.2		
	Pap-smear	52.2		
	Mental healthcare	35.8		
	Geriatric care	30.2		
	Allergy/Dermatology	6.9		
	Osteoporosis	10.8		
	Immigrants	1.7		
	Travellers	25.9		
	Other special consultation hours	7.3		
Patient information material	Sum score of the 6 underlying items		3.32	1.27
*The practice has the following communication channels/materials:*	Leaflets	85.8		
	Leaflets in different languages	3.0		
	Website	76.7		
	Patient letters	86.6		
	Leaflet on complaint procedure	79.3		
	Other patient informational material	0.4		

#### Socio-demographic profile of the patients’ area of residence

Statistics Netherlands provided national census data at the four-digit postcode level from the year 2013 [18]. The data collected included the total population, the number of males and females in different age categories, one-person households, non-Western immigrants (at least one parent born in Africa, Latin America and/or Asia), low-income households (households with a purchasing power of less than <€9250 a year) and urbanization level of patients’ area, divided into five categories. Additionally, status score was obtained from the Netherlands Institute for Social Research. Status score is an indicator of the socioeconomic status of an area [19]. The area characteristics were selected as explanatory variables of the utilization of general practice care, because they are available at the four-digit postcode level and are important determinants of healthcare use [20].

#### Outcome measures

The first outcome variable was the actual GP utilization rate per listed patient per practice, which was the sum of all declared consultations, such as consultations (including by email), telephone consultations, home visits and consultations involving minor surgery. The second outcome variable was the estimated GP utilization rate per listed patient per practice based on the socio-demographic profile of patients’ area. The third outcome variable was the difference between the actual GP utilization rate and the estimated rate per listed patient per practice. This continuous variable was analysed as such and also defined into categories by ranking. The first 40 scores made up the category ‘low utilization’, in which the actual utilization rate was lower than the estimated rate. The final 40 scores made up the category ‘high utilization’, in which the actual utilization rate was higher than the estimated rate.

#### Data analyses

First, descriptive analyses were calculated to describe the sample and the explanatory variables at practice level.

Second, linear regression was used to analyse whether practice organization characteristics could explain the actual utilization rate aggregated at practice level.

Third, on the same data but now aggregated at the four-digit postcode level a linear regression model was estimated for utilization on socio-demographic characteristics of patients’ area of residence. Only four-digit postcode areas with at least 100 listed patients contributed to the model. Predictions were made for all four-digit postcodes in the dataset. Subsequently, a utilization rate was calculated for each general practice as a weighted mean of the predictions of the patients’ postcodes. In the model the next socio-demographic predictors were used: the percentages of male and female in seven different age categories (0–4, 5–14, 15–24, 25–39, 40–64, 65–74, 75 and older), the percentages of one-person households, non-Western immigrants, low-income households, urbanization level (which was dummy-coded) and status score.

Fourth, this estimated utilization rate per practice was compared with the actual utilization rate. Linear regression was used to analyse whether the difference could be explained by practice organization characteristics, on aggregated data at practice level. The explained variance of the linear regression models was given by R-squared.

In addition, t-tests and proportion-tests were conducted to contrast practices with the highests positive difference with those with the highest negative difference on practice characteristics.

Analyses were conducted using Stata version 14.1.

## Results

### Sample characteristics and practice organization measures

Table 1 presents the mean of the five practice organization measures and the percentages of the underlying items. Scheduled consultation hours, minor surgery and an emergency line are the three most frequent practice organization characteristics in general practice. Table 2 shows the distribution of the practice organization characteristics of the general practices in the sample (*n* = 232). The mean number of GPs per practice is 2.32.

**Table 2:** Distribution of the selected practice characteristics for the total sample of general practices (*n* = 232)

Practice characteristics	N	%
Solo practices	88	37.9
Dual practices	91	39.2
Group practices	53	22.8
Practices in rural areas	37	16.0
Practices in low urbanized areas	42	18.1
Practices in moderately urbanized areas	47	20.3
Practice in highly urbanized areas	45	19.4
Practices in very highly urbanized areas	61	26.3
Support staff per practice	N	%
Doctor’s assistant	213	91.8
Practice nurse somatic disorders	85	36.6
Practice nurse mental disorders	146	62.9
Pharmacist	9	3.9
Other medical providers	25	10.8
Other non-medical providers	54	23.2
GP characteristics per practice	M	SD
GPs	2.32	1.49
FTE GP	1.62	1.12
Female GP	1.09	1.05
GPs younger than 40 years old	0.47	0.73
GPs 40–55 years old	1.18	1.10
GPs 56–65 years old	0.63	0.83
GPs 65 years or older	0.05	0.22
Self-employed GPs	1.84	1.29
GPs employed by GP	0.26	0.50
Locums	0.17	0.43

### Mean actual utilization rate per practice

The regression coefficients for the different practice organization characteristics on the mean actual utilization rate per practice are depicted in Table 3, *F* (17, 206) = 3.12; *p* = 0.0001). The results show that three practice characteristics are statistical significant positive predictors of the mean actual utilization rate, namely ‘disease management programme’, ‘the presence of other medical providers in practice’ and ‘dual practices’. The model explains 20.5% of the variation in the dependent variable.

**Table 3:** Model estimates for the regression of mean actual utilization rate on practice characteristics. (Data aggregated on practice level, *n* = 224)

	B	*P*-value	Lower bound 95% CI	Upper bound 95% CI
Practice organization measures				
Medical service profile	0.048	0.33	−0.049	0.145
Consultation profile	0.070	0.22	−0.041	0.182
Accessibility	0.156	0.05	−0.001	0.312
Disease management	0.048^a^	0.04	0.001	0.094
Patient information material	−0.074	0.23	−0.196	0.048
Human resources in practice				
Doctor’s assistant	−0.456	0.18	−1.125	0.212
Practice nurse somatic disorders	−0.265	0.07	−0.553	0.024
Practice nurse mental disorders	0.047	0.70	−0.188	0.281
Pharmacist	0.145	0.60	−0.403	0.694
Other medical providers	0.618^a^	0.00	0.298	0.938
Other non-medical providers	−0.141	0.24	−0.375	0.094
Type of practice; reference is solo practice				
Dual practices	0.283^a^	0.02	0.040	0.525
Group practices	0.141	0.32	−0.136	0.419
GP characteristics per practice				
Percentage of female GPs	0.003	0.10	−0.001	0.006
Percentage of GPs younger than 40 years	0.002	0.28	−0.002	0.006
Percentage of self-employed GPs	−0.001	0.66	−0.005	0.003
Number of patients per FTE GP	0.000	0.82	−0.000	0.000

^a^*p* < 0.05. *CI* Confidence interval, R^2^ = 20.5%; Adjusted R^2^ = 13.9%

### Mean estimated utilization rate per postcode

The mean estimated utilization rate was calculated at the four-digit postcode level using linear regression analysis. The results of the linear regression analysis are given in Table 4, with the mean utilization rate as dependent variable and the socio-demographic characteristics of patients’ area as independent variables, *F* (21, 871) = 14.33, *p* < 0.0001. The model explains 25.7% of the variation in the dependent variable. Seven predictors are statistical significant. The predictors ‘females of 75 years and older’, ‘persons in a low-income household’, ‘low urbanized areas’, ‘moderately urbanized areas’, ‘strongly urbanized areas’ and ‘very strongly urbanized areas’ have a positive association. The predictor ‘one-person households’ has a negative association with the dependent variable.

**Table 4:** Model estimates for the regression of mean utilization rate on sociodemographic characteristics of four-digit postcode areas (data aggregated on four-digit postcode area, *n* = 893)

	B	P-value	Lower bound 95% CI	Upper bound 95% CI
Percentage gender age categories; reference category is proportion of males 25–39 years old				
Males 0–4 years old	− 0.085	0.23	−0.225	0.055
Females 0–4 years old	0.028	0.68	−0.106	0.162
Males 5–14 years old	−0.014	0.74	−0.099	0.070
Females 5–14 years old	−0.069	0.14	−0.161	0.023
Males 15–24 years old	− 0.042	0.38	−0.135	0.052
Females 15–24 years old	−0.028	0.38	−0.092	0.035
Females 25–39 years old	0.035	0.53	−0.074	0.145
Males 40–64 years old	−0.024	0.55	−0.102	0.054
Females 40–64 years old	0.044	0.14	−0.014	0.103
Males 65–74 years old	0.053	0.35	−0.058	0.163
Females 65–74 years old	−0.064	0.24	−0.171	0.043
Males 75 years or older	0.096	0.10	−0.019	0.212
Females 75 years or older	0.080^a^	0.04	0.005	0.156
Percentage of one-person households	−0.012^a^	0.03	−0.023	− 0.001
Percentage of non-Western immigrants	0.005	0.12	−0.001	0.012
Percentage of people in low-income households	0.030^a^	0.00	0.012	0.047
Status score	0.045	0.34	−0.048	0.139
Degree of urbanization; reference category is rural				
Low urbanisation	0.387^a^	0.00	0.191	0.583
Moderately urbanised	0.302^a^	0.00	0.099	0.505
Strongly urbanised	0.381^a^	0.00	0.161	0.600
Very strongly urbanised	0.345^a^	0.02	0.059	0.631

^a^*p* < 0.05. CI=Confidence interval, R^2^ = 25.7%, Adjusted R^2^ = 23.9%

### Difference between actual and postcode-based estimated utilization rate

The results of the linear regression analysis are shown in Table 5, with the difference between the actual and the estimated utilization rate as dependent variable and practice organization characteristics as independent variables, *F*(17, 206) = 2.89, *p* = 0.0002. The results show four practice characteristics with a statistical significant positive influence on the difference between the mean actual utilization rate and the mean estimated utilization rate, namely ‘percentage of female GPs’, ‘disease management programme’, ‘the presence of other medical providers in practice’ and ‘dual practices’. The model explains 19.3% of the variation in the dependent variable.

**Table 5:** Model estimates for the regression of difference between the actual and estimated utilization rate on practice characteristics (data aggregated on practice level, *n* = 224)

	B	P-value	Lower bound 95% CI	Upper bound 95% CI
Practice organization measures				
Medical service profile	0.055	0.24	−0.038	0.148
Consultation profile	0.081	0.14	−0.026	0.188
Accessibility	0.107	0.16	−0.043	0.257
Disease management	0.051^a^	0.03	0.007	0.095
Patient information material	−0.052	0.38	−0.170	0.065
Human resources in practice				
Doctor’s assistant	−0.500	0.13	−1.142	0.141
Practice nurse somatic disorders	−0.269	0.06	−0.546	0.008
Practice nurse mental disorders	0.000	1.00	−0.225	0.225
Pharmacist	0.183	0.49	−0.343	0.710
Other medical providers	0.463^a^	0.00	0.156	0.770
Other non-medical providers	−0.139	0.23	−0.364	0.087
Type of practice; reference is solo practice				
Dual practices	0.253^a^	0.03	0.021	0.486
Group practices	0.138	0.31	−0.129	0.404
GP characteristics per practice				
Percentage of female GPs	0.003^a^	0.04	0.0001	0.007
Percentage of GPs younger than 40 years	0.002	0.27	−0.002	0.006
Percentage of self-employed GPs	0.000	0.89	−0.004	0.004
Number of patients per FTE GP	0.000	0.42	0.000	0.000

^a^*p* < 0.05. CI=Confidence interval, R^2^ = 19.3%; Adjusted R^2^ = 12.6%

In addition to the analysis of practice organization characteristics on the difference between the actual utilization rate and the postcode-based estimated utilization rate, we also analysed ‘the postcode-based estimated utilization rate’ as a predictor for the mean utilization rate per practice. The results show that on its own, the ‘postcode-based estimated utilization rate’ (*B* = 0.93) accounts for 9.2% of the variation in the mean utilization rate per practice.

### T-tests and proportion tests on low versus high utilization practices

Table 6 illustrates the distribution of practice characteristics for the total sample of general practices and for the general practices divided into two categories: low utilization and high utilization. Table 6 also shows the results of the t-tests and the proportion-tests between the practices with the 40 highest positive and the 40 highest negative differences. Compared to practices with high utilization, practices with low utilization are significantly more often solo practices, less often dual practices, have lower numbers of GPs, female GPs, as well as GPs younger than 40 years old. They also score significantly lower on the practice organization measure ‘consultation profile’.

**Table 6:** Differences on practice organization characteristics between the 40 lowest and 40 highest scoring practices on the difference between mean actual utilization rate and the postcode-based estimate

	Total GP practices in sample (*N* = 232)		Practices with utilization lower than predicted *N* = 40		Practices with utilization higher than predicted *N* = 40		Results t-tests/pr-tests between low and high utilization practices
	Mean	*SD*	Mean	*SD*	Mean	*SD*	*P-value*
GP characteristics per practice							
GPs per practice	2.32	*1.50*	**1.73**	*0. 88*	**2.40**	*1.60*	*0.02* ^a^
Female GPs per practice	1.09	*1.05*	**0.60**	*0.60*	**1.28**	*1.13*	0.00**
GPs younger than 40 years	0.47	*0.73*	**0.20**	*0.41*	**0.53**	*0.72*	*0.02* ^a^
GPs 40–55 years	1.17	*1.10*	0.98	*1.00*	1.13	*0.94*	*0.49*
GPs 56–65 years	0.63	*0.83*	0.50	*0.60*	0.63	*0.87*	*0.46*
GPs 65 years or older	0.05	*0.22*	0.05	*0.22*	0.13	*0.34*	*0.24*
Self-employed GPs per practice	1.84	*1.29*	1.38	*0.81*	1.68	*1.00*	*0.14*
GPs employed by GPs per practice	0.26	*0.51*	0.18	*0.39*	0.30	*0.61*	*0.28*
Locums per practice	0.17	*0.43*	0.18	*0.39*	0.25	*0.44*	*0.42*
Missing	0.05	*–*	–	*–*	0.17	*–*	*–*
FTE GP per practice	1.62	*1.12*	1.28	*0.70*	1.59	*1.14*	*0.15*
Practice characteristics							
List size per practice	3671	*2384*	2767	*909*	3502	*2233*	*0.06*
Utilization rate per patient	4.05	*0.75*	**3.18**	*0.35*	**5.21**	*0.63*	0.00**
Solo practices	0.38	*0.49*	**0.60**	*0.50*	**0.30**	*0.46*	0.00**
Dual practices	0.39	*0.49*	**0.30**	*0.46*	**0.58**	*0.50*	*0.01* ^a^
Group practices	0.23	*0.42*	0.10	*0.30*	0.13	*0.34*	*0.72*
Urbanization	3.22	*1.42*	2.90	*1.50*	3.23	*1.39*	*0.32*
Practice organization measures							
Medical service profile	2.99	*1.50*	2.75	*1.43*	3.35	*1.55*	*0.08*
Consultation profile	4.11	*1.54*	**4.03**	*1.54*	**4.73**	*1.40*	*0.04* ^a^
Accessibility	1.44	*0.75*	1.33	*0.80*	1.63	*0.67*	*0.07*
Disease management	4.18	*3.25*	3.75	*2.89*	5.18	*3.74*	*0.06*
Patient information material	3.32	*1.27*	3.30	*1.27*	3.53	*1.20*	*0.42*
Support staff per practice							
Doctor’s assistant	0.92	*0.27*	0.90	*0.30*	0.95	*0.22*	*0.40*
Practice nurse somatic disorders	0.37	*0.48*	0.38	*0.49*	0.48	*0.51*	*0.37*
Practice nurse mental disorders	0.63	*0.48*	0.43	*0.50*	0.63	*0.49*	*0.24*
Pharmacist	0.04	*0.20*	0.03	*0.16*	0.00	*0.00*	*0.31*
Other medical providers	*0.11*	0.31	0.05	*0.22*	0.18	*0.39*	*0.08*
Other non-medical providers	0.23	*0.42*	0.25	*0.44*	0.20	*0.41*	*0.39*

^a^The score for low utilization practices is significantly different from high utilization practices; *P* < 0.05; **P < 0.01

Bold The numbers in bold are practice characteristics which differ significantly between low and high utilization practices

## Discussion

The present study, is a first exploration on the extent to which differences between the actual utilization rate and the estimated rate based on a limited set of socio-demographic variables can be related to practice organization characteristics. The socio-demographic profile of the patients’ area of residence accounted for approximately 26% of the actual GP utilization rate at the four-digit postcode level. The investigated practice organization characteristics accounted for approximately 20% of the actual GP utilization rates. Three practice organization characteristics were statistically significant. The mean actual utilization rate per patient increased by 0.6 contacts when a ‘other medical provider’ was present in the general practice, increased by 0.3 when the practice was a dual practice instead of a solo practice, and increased by 0.05 with every added disease management programme.

Practice organization characteristics accounted for 19% of the difference between the actual. GP utilization rates and the estimated rates based on the socio-demographic predictors. The practice characteristics mentioned above were also statistical significant here: ‘the presence of other medical providers’, ‘dual practice’ and ‘the availability of a disease management programme’. Additionally, the employment of female GPs significantly increased GP utilization rates. Every extra percentage of female GPs added 0.003 to the utilization rate per patients. Together with the results of the t-tests and proportion-tests, these results indicate lower utilization rates than estimated for solo practices, with fewer GPs, female GPs, GPs younger than 40 years, and for practices which offer fewer consultation types. The actual utilization rates of these practices are lower than expected based on the socio-demographic profile of the practice population.

The present study, cannot comment on the quality of general practice care for high or low utilization practices. Huygen et al. (1992) found that patients from a doctor with an integrated practice style, which is regarded as good quality of care, have a better health and visited their doctor less frequently. Moreover, those doctors kept the referrals to a specialist to a minimum [7]. So, low utilization practices can provide good quality of care. Low utilization practices may also keep the quality of care high by the employment of more experienced GPs who need fewer follow-up consultations. The age of the GP may be seen as an indicator for experience. We found a significant lower percentage of GPs younger than 40 years in practices with low utilization. However, Kersnik found that frequent attenders were more likely to visit an experienced GP and found no differences in the age of the GP between frequent attenders and infrequent attenders. In further research, the relationship between quality of care, the experience of GPs and high or low utilization should be investigated.

Provider characteristics interact with patient characteristics to influence utilization of care [21]. One important result of our study is that a higher utilization rate is found more often in practices with a higher number of female GPs. In a Dutch study, Bensing found gender differences in practice style: female GPs spend more time with their patients, female patients tend to choose female GPs and female GPs see more gynecologic problems and consults for family planning [22]. Also, Majeed et al. (1994) found higher cervical smear uptake rates in practices with a female partner and in larger practices. Thus, hiring female GPs will probably attract more female patients, who are more frequent attenders in general practice care for female related health problems which result in higher practice consultation rates. However, in a study of Kernsik, it was found that frequent attenders were more likely to visit a male GP. So, in future research the relationship between the use of GP care and the gender of the GP has to be further investigated.

High or low utilization of general practice care can be seen as an indicator for the accessibility of general practice care, because utilization of care is influenced by the availability and accessibility of health care services, next to health status and health related behaviour [23]. Accessibility of health care is a multidimensional concept. Elements such as geographical accessibility, availability, affordability, acceptability and accommodation can be distinguished [24–26]. A Danish study by Heje on the accessibility of general practice care found that patients experienced better accessibility in solo practices, with a short patient list and with a few employees [27]. We found that low utilization practices are more often solo practices with less FTE GP. In the study by Heje, patients may have reported better accessibility of care in these smaller solo practices because they experienced more continuous care in the doctor-patient relationship [28]. Moreover, a better perceived accessibility of care may not always lead to high utilization rates. However, we found no influence of our accessibility measure. Our accessibility measure only existed of three accessibility issues and was not based on a theoretical framework. Thus, the relationship between the accessibility measure and utilization rates should be interpreted with caution. In further research, the accessibility of low and high utilization practices should be further investigated.

To the best of our knowledge, our study is one of the first to assess low utilization or high utilization of general practice care using an SAE method, which is a relatively easy, robust and inexpensive method. Our findings indicate that high utilization was found more often in general practices that employ other medical providers and that offer a disease management programme. Our assumption is, that larger practices have more human resources and are technically better equipped to provide consultations for minor surgery or for specific diseases, resulting in higher utilization rates than solo practices. GPs in practices with high utilization rates and probably a high workload may keep their practices accessible by task shifting, task delegation, work efficiency and shorter patient time, as was concluded by Van den Berg for practices with a higher workload in the Netherlands [29, 30]. However, our study is a first exploration and future research is needed to investigate the exact influence of practice organization characteristics and their interactions.

### Strengths and limitations

We would have preferred to use a crossed-effects multilevel model with practices as one source of level two variation and four-digit postcodes as the other. However, this was not feasible due to the long estimation time, because of the high number of level 2 units: 232 general practices and 893 four-digit postcode areas.

Our results are based on three large datasets of routinely registered data. One contains data on the characteristics of all the practices and GPs in the Netherlands, the other contains health record data of approximately 1 million patients who are representative of the Dutch population regarding age and gender, and the last dataset contains census data which are routinely updated by Statistics Netherlands. However, these datasets entails also limitations. GP record data may be registered with some errors. The practice characteristics are expected to be updated by the GPs yearly, however it is not known for sure whether a change in the practice organization is also being registered. Our 232 general practices are not a random sample, but comes from a historical grown register for which practices were selected. Thus, the representativity of our findings is unclear.

A strength of our study was the large number of practice characteristics, including the five practice organization measures, that we have investigated as potential factors of low or high utilization. Third, we used a powerful alternative to costly designs, i.e. the small area estimation method [31, 32]. This method has been applied in several policy areas around the world, including health. In previous research, we calculated estimates on the need for general practice care in all the local areas in the Netherlands [33].

However, the use of the SAE method also has a disadvantage. The SAE method gives the expected value for an area based on the socio-demographic predictors included in the model and not the real value for the construct. Therefore, results which are based on SAE measures are” ..usually at pains to stress that it is reporting estimates all with a degree of uncertainty and not a direct measure of the construct of concern” [34]. This degree of uncertainty may be enhanced by the fact that the model incorporates the mean value of a socio-demographic predictor for an area, such as the percentage of low income households, while the patients belonging to a practice are a selection from this area and may in fact belong to a higher income category.

## Conclusions

The contributors to the utilization of general practice care are manifold and can be found at different levels. We found contributors at the four-digit postcode level and at the general practice level. The presence of other medical providers in the practice, the presence of female GPs and the number of disease management programmes influenced the difference between the actual utilization rate and the estimated rate based on the socio-demographic profile of an area. Our findings provide stakeholders who are involved with the organization of general practice care with useful insight into the practice organization characteristics that influence high or low utilization, in order to help them reflect on the organization and utilization of general practice care.

## Abbreviations

FTE GP: Full-time equivalent general practitioner
GP: General practitioner
OHCP: Other healthcare provider
SAE: Small area estimation
